# A study of the risk factors for phlebitis in patients stratified using the acute physiology and chronic health evaluation II score and admitted to the intensive care unit: A *post hoc* analysis of the AMOR-VENUS study

**DOI:** 10.3389/fmed.2022.965706

**Published:** 2022-12-05

**Authors:** Yuki Kishihara, Hideto Yasuda, Takashi Moriya, Masahiro Kashiura, Midori Koike, Yuki Kotani, Natsuki Kondo, Kosuke Sekine, Nobuaki Shime, Keita Morikane, Takayuki Abe

**Affiliations:** ^1^Department of Emergency and Critical Care Medicine, Jichi Medical University Saitama Medical Center, Saitama, Japan; ^2^Department of Clinical Research Education and Training Unit, Keio University Hospital Clinical and Translational Research Center (CTR), Tokyo, Japan; ^3^Department of Intensive Care Medicine, Kameda Medical Center, Chiba, Japan; ^4^Department of Intensive Care Medicine, Chiba Emergency Medical Center, Chiba, Japan; ^5^Department of Medical Engineer, Kameda Medical Center, Chiba, Japan; ^6^Department of Emergency and Critical Care Medicine, Graduate School of Biomedical and Health Sciences, Hiroshima University, Hiroshima, Japan; ^7^Division of Clinical Laboratory and Infection Control, Yamagata University Hospital, Yamagata, Japan; ^8^Biostatistics, Clinical and Translational Research Center, Keio University School of Medicine, Tokyo, Japan; ^9^School of Data Science, Yokohama City University, Kanagawa, Japan

**Keywords:** APACH II score, catheter, critically ill patient, risk factors, intensive care unit (ICU), peripheral, phlebitis

## Abstract

**Introduction:**

Peripheral intravascular catheters (PIVCs) are inserted in most patients admitted to the intensive care unit (ICU). Previous research has discussed various risk factors for phlebitis, which is one of the complications of PIVCs. However, previous studies have not investigated the risk factors based on the patient’s severity of illness, such as the Acute Physiology and Chronic Health Evaluation (APACHE) II score. Different treatments can be used based on the relationship of risk factors to the illness severity to avoid phlebitis. Therefore, in this study, we investigate whether the risk factors for phlebitis vary depending on the APACHE II score.

**Materials and methods:**

This study was a *post hoc* analysis of the AMOR-VENUS study involving 23 ICUs in Japan. We included patients with age ≥ 18 years and consecutive admissions to the ICU with PIVCs inserted during ICU admission. The primary outcome was phlebitis, and the objective was the identification of the risk factors evaluated by hazard ratio (HR) and 95% confidence interval (CI). The cut-off value of the APACHE II score was set as ≤15 (group 1), 16–25 (group 2), and ≥26 (group 3). Multivariable marginal Cox regression analysis was performed for each group using the presumed risk factors.

**Results:**

A total of 1,251 patients and 3,267 PIVCs were analyzed. Multivariable marginal Cox regression analysis reveals that there were statistically significant differences among the following variables evaluated HR (95%CI): (i) in group 1, standardized drug administration measures (HR, 0.4 [0.17–0.9]; *p* = 0.03) and nicardipine administration (HR, 2.25 [1.35–3.75]; *p* < 0.01); (ii) in group 2, insertion in the upper arm using the forearm as a reference (HR, 0.41 [0.2–0.83]; *p* = 0.01), specified polyurethane catheter using polyurethane as a reference (HR, 0.56 [0.34–0.92]; *p* = 0.02), nicardipine (HR, 1.9 [1.16–3.12]; *p* = 0.01), and noradrenaline administration (HR, 3.0 [1.52–5.88]; *p* < 0.01); (iii) in group 3, noradrenaline administration (HR, 3.39 [1.14–10.1]; *p* = 0.03).

**Conclusion:**

We found that phlebitis risk factors varied according to illness severity. By considering these different risk factors, different treatments may be provided to avoid phlebitis based on the patient’s severity of illness.

## Introduction

Most patients admitted to the intensive care unit (ICU) are inserted with peripheral intravascular catheters (PIVCs). However, various adverse events associated with the insertion of PIVCs are known, including hematoma, skin inflammation associated with drug leakage, and phlebitis (1). Previous studies have reported the occurrence of phlebitis at a rate of 7.5% per catheter (2). Phlebitis may be considered a major complication since mild phlebitis can cause pain and anxiety, while severe phlebitis can cause skin necrosis and infective endocarditis (3–5).

As discussed in a previous study, risk factors for phlebitis in patients admitted to the ICU include patients’ body mass index (BMI), ICU characteristics, medical staff inserting the catheter, insertion site, and type of drugs administered (6). Furthermore, the risk of phlebitis may be higher in patients with particularly severe conditions, as represented by a high Acute Physiology and Chronic Health Evaluation (APACHE) II score (7). The APACHE II score is commonly used worldwide as a determination of severity and predictor of prognosis in patients admitted to the ICU. Therefore, it seems reasonable to use the APACHE II score to stratify severity in studies of ICU patients. However, previous studies have not investigated the risk factors for phlebitis stratified by APACHE II severity in patients admitted to the ICU.

If the risk factors for phlebitis vary according to the patient’s severity of illness, a different treatment may be provided to avoid phlebitis by considering these risk factors, particularly for critically ill patients who are expected to be more affected by phlebitis. Therefore, this study aimed to investigate whether the different risk factors for phlebitis vary depending on the patient’s severity of illness as classified using the APACHE II score.

## Materials and methods

### Study design

This study was a *post hoc* analysis of the AMOR-VENUS database from a previous prospective multicenter cohort study that involved 22 institutions and 23 ICUs in Japan between January 1, 2018 and March 31, 2018 (2). The AMOR-VENUS study was registered at the University Hospital Medical Information Network Clinical Trials Registry under the Japanese clinical trial registry (registration number: UMIN000028019) and was approved by the institutional review board or medical ethics committee of each educational institution. This study is intended to investigate the epidemiology of PIVC-induced phlebitis in intensive care and its risk factors. A new ethical review was waived for this study since the approval for the AMOR-VENUS study included *post hoc* analysis using the AMOR-VENUS database. This study was based on Strengthening the Reporting of Observational Studies in Epidemiology (STROBE) guidelines (8). (Supplementary Table 1 in Supplementary material).

### Patients

The inclusion criteria of the AMOR-VENUS study database were: (1) age ≥ 18 years; and (2) consecutive admissions to the ICU with PIVCs inserted during ICU admission during the study enrollment period. The details of the inclusion and exclusion criteria are described in the AMOR-VENUS study (2). In addition, the exclusion criteria in this study were: (1) PIVCs inserted outside the ICU; (2) missing APACHE II score data; and (3) use of unclassifiable catheter material. Since detailed drug information is necessary for the analysis of this study, PIVCs inserted outside the ICU were excluded.

### Data collection

The following data were collected in this study: patient characteristics (age, sex, height, weight, BMI, Charlson Comorbidity Index, APACHE II score, type of ICU admission, ICU admission category, presence of sepsis at ICU admission, and presence of mechanical ventilation), provision of standardized drug administration measures in the ICU, PIVC characteristics (medical staff inserting the catheter, insertion site, catheter materials, catheter gauge, duration of catheter dwell), drugs administered via PIVCs during ICU stay (albumin, amiodarone, dobutamine, fat, fentanyl, heparin, magnesium, meropenem, midazolam, nicardipine, nitroglycerin, noradrenaline, potassium, and vancomycin), ICU mortality, and outcome of phlebitis (6). The APACHE II score was calculated using the worst value after 24 h of hospitalization. Phlebitis was defined using the Phlebitis Scale developed by the Infusion Nurses Society (9). Detailed information on the definition and methods of phlebitis evaluation has been described in the AMOR-VENUS study and Supplementary Tables 2, 3.

### Study outcomes

The primary outcome was phlebitis, and the primary objective was the identification of the risk factors for phlebitis evaluated by hazard ratio (HR).

### Statistical methods

Continuous variables are presented using means and standard deviations (SD) or median and interquartile range (IQR) and were analyzed using the analysis of variance or Kruskal–Wallis test. Categorical variables are presented using absolute counts and percentages (%) and were analyzed using Fisher’s exact test or Pearson’s chi-square test.

The cut-off value of the APACHE II score for the classification of patients by the severity of illness was set. A previous study used the spline curves to set the cut-off values as ≤15 (group 1), 16–25 (group 2), and ≥26 (group 3) (6). These values were also used for the analysis in this study. Subsequently, univariate and multivariable marginal Cox regression analyses were used for each of the patient strata stratified using the APACHE II score to assess the association between the time of phlebitis occurrence and presumed risk factors, since there were within-patient and within-institution correlations between the catheters. The time of PIVC insertion in the ICU was defined as the time zero of the marginal Cox regression model, as well as the occurrence of phlebitis, removal of PIVC, or the time of ICU discharge if the patient left the ICU with the PIVC inserted as censoring. Considering a previous study (6), the following presumed risk factors for phlebitis were extracted: patient characteristics (age, sex, BMI, and APACHE II score), provision of standardized drug administration measures in the ICU, PIVC characteristics (medical staff inserting the catheter, insertion site, and catheter materials), and drugs administered via PIVCs during ICU stay (amiodarone, heparin, nicardipine, nitroglycerin, noradrenaline, and vancomycin). Based on the classification of the World Health Organization and considering that this was primarily composed of an Asian population, BMI was categorized into three groups as follows: ≤ 18.5, 18.5–25, and ≥ 25 (10, 11). The drugs included in this model as binary data were based on a previous study (6) and selected with the following considerations: (1) administered at a percentage more frequently than 5% of all PIVCs, (2) incidence of phlebitis was ≥ 1%, (3) calculated p values in a previous study using multivariable marginal Cox regression analysis for phlebitis were >0.1, and (4) clinical significance. Since the distribution of the missing data was not random, imputation was not performed, and only complete cases were analyzed. Effect estimates were described using HR and a 95% confidence interval (CI). The analyses were performed using EZR version 1.38 and SAS Studio (SAS Inc., Cary, NC), and *p* < 0.05 was considered statistically significant by a two-sided test.

## Results

**FIGURE 1:**
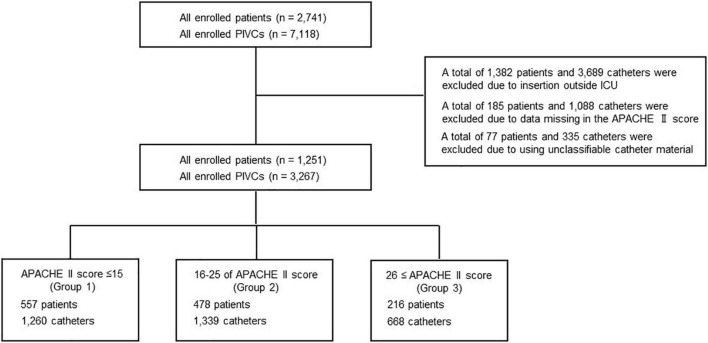
Flowchart depicting the screening and enrolment process within the study. APACHE, acute physiology and chronic health evaluation; ICU, intensive care unit; PIVC, peripheral intravascular catheter.

A total of 2,741 patients were included and 7,118 PIVCs were inserted (Figure 1). Meanwhile, 1,382 patients and 3689 PIVCs were excluded due to insertion outside the ICU; 185 patients and 1,088 catheters were excluded due to missing APACHE II score data; and 77 patients and 335 catheters were excluded due to the use of unclassifiable catheter material. Finally, a total of 1,251 patients and 3,267 PIVCs inserted were analyzed. Moreover, 557 patients (44.5%) and 1,260 catheters (38.6%) were categorized in group 1 (APACHE II score ≤ 15); 478 patients (38.2%) and 1,339 catheters (41.0%) were in group 2 (16–25 of the APACHE II score); and 216 patients (17.3%) and 668 catheters (20.4%) were in group 3 (APACHE II score ≥ 26) (Tables 1, 2).

**TABLE 1:** Patient characteristics at ICU admission stratified using the APACHE II score.

Variables	Overall *n* = 1,251	Group 1 (APACHE II score ≤ 15) *n* = 557 (44.5%)	Group 2 (16–25 of APACHE II score) *n* = 478 (38.2%)	Group 3 (APACHE II score ≥ 26) *n* = 216 (17.3%)	*p* value
Age, mean (SD), years	68.5 (15.2)	64.0 (16.4)	72.4 (13.0)	71.4 (13.1)	<0.01
Men (n,%)	819 (63.2)	382 (65.7)	292 (58.9)	145 (66.5)	0.04
Body height^a^, mean (SD), cm	160.8 (9.8)	162.7 (9.7)	158.9 (9.6)	160.0 (9.6)	<0.01
Body weight, mean^b^ (SD), kg	59.6 (14.7)	62.6 (16.0)	56.3 (13.4)	58.9 (12.5)	<0.01
BMI^b^, mean (SD)	22.9 (4.4)	23.5 (4.7)	22.2 (4.2)	22.9 (3.9)	<0.01
Charlson comorbidity index, mean (SD)	4.4 (2.6)	3.7 (2.4)	4.9 (2.4)	5.2 (2.9)	<0.01
**Type of admission to ICU (n,%)**					
Elective surgical	352 (28.1)	254 (45.6)	85 (17.8)	13 (6.0)	<0.01
Emergency surgical	205 (16.4)	78 (14.0)	95 (19.9)	32 (14.8)	<0.01
Medical	694 (55.5)	225 (40.4)	298 (62.3)	171 (79.2)	<0.01
**ICU admission category^c^ (n,%)**					
Cardiology	449 (35.9)	230 (41.3)	156 (32.6)	63 (29.2)	<0.01
Pulmonary	181 (14.5)	63 (11.3)	80 (16.7)	38 (17.6)	<0.01
Gastrointestinal	160 (12.8)	76 (13.6)	63 (13.2)	21 (9.7)	<0.01
Neurology	182 (14.6)	79 (14.2)	73 (15.3)	30 (13.9)	<0.01
Trauma	51 (4.1)	33 (5.9)	14 (2.9)	4 (1.9)	<0.01
Urology	16 (1.3)	10 (1.8)	5 (1.0)	1 (0.5)	0.02
Gynecology	11 (0.9)	9 (1.6)	2 (0.4)	0 (0)	<0.01
Skin/tissue	22 (1.8)	10 (1.8)	10 (2.1)	2 (0.9)	0.05
Others	63 (5.0)	32 (5.7)	20 (4.2)	11 (5.1)	<0.01
**Sepsis at ICU admission (n,%)**					
Sepsis	85 (6.8)	19 (3.4)	37 (7.7)	29 (13.4)	0.06
Septic shock	128 (10.2)	17 (3.1)	59 (12.3)	52 (24.1)	<0.01
Mechanical ventilation within 24 h of admission to the ICU (n,%)	633 (50.6)	180 (32.3)	283 (59.2)	170 (78.7)	<0.01
ICU mortality (n,%)	63 (5.0)	5 (0.9)	18 (3.8)	40 (18.5)	<0.01
Phlebitis (n,%)	97 (7.8)	37 (6.6)	37 (7.7)	23 (10.6)	0.2

APACHE, acute physiology and chronic health evaluation; BMI, body mass index; ICU, intensive care unit; PIVC, peripheral intravascular catheter; SD, standard deviation. Missing data: a, *n* = 1 (0.08%); b, *n* = 2 (0.2%); c, *n* = 7 (0.6%).

**TABLE 2:** All PIVC characteristics during insertion stratified using the APACHE II score.

Variables	Overall *n* = 3,267	Group 1 (APACHE II score ≤ 15) *n* = 1,260 (38.6%)	Group 2 (16–25 of APACHE II score) *n* = 1,339 (41.0%)	Group 3 (APACHE II score ≥ 26) *n* = 668 (20.4%)	*p* value
Provision of standardized drug administration measures in the ICU (n,%)	3,219 (98.5)	1,226 (97.3)	1,325 (99.0)	668 (100)	<0.01
**Medical staff inserting the catheter^a^ (n,%)**					
Doctor	279 (8.5)	93 (10.6)	117 (10.4)	69 (11.7)	<0.01
Nurse	2313 (70.8)	788 (89.4)	1,005 (89.5)	520 (88.3)	<0.01
Medical technologist	1 (0.03)	0 (0)	1 (0.1)	0 (0)	−**
**Insertion site (n,%)**					
Forearm	1,752 (53.6)	709 (56.4)	717 (54.2)	326 (49.1)	0.03
Upper arm	344 (10.5)	95 (7.6)	148 (11.2)	101 (15.2)	<0.01
Elbow	152 (4.7)	54 (4.3)	62 (4.7)	36 (5.4)	<0.01
Wrist	157 (4.8)	66 (5.3)	69 (5.2)	22 (3.3)	<0.01
Hand	486 (14.9)	232 (18.5)	170 (12.8)	84 (12.7)	<0.01
Lower leg	218 (6.7)	64 (5.1)	103 (7.8)	51 (7.7)	<0.01
Dorsal foot	135 (4.1)	36 (2.9)	55 (4.2)	44 (6.6)	0.13
**Catheter material (n,%)**					
PEU-Vialon^§^ *	1040 (31.8)	456 (36.2)	412 (30.8)	172 (25.7)	<0.01
Polyethylene	975 (29.8)	385 (30.6)	443 (33.1)	147 (22.0)	<0.01
Tetrafluoroethylene	1252 (38.3)	419 (33.3)	484 (36.1)	349 (52.2)	<0.01
**Catheter gauge^b^ (n,%)**					
14G	1 (0.03)	1 (0.1)	0 (0)	0 (0)	−**
16G	72 (2.2)	38 (3.1)	29 (2.2)	5 (0.8)	<0.01
18G	87 (2.7)	58 (4.7)	19 (1.4)	10 (1.5)	<0.01
20G	818 (25.0)	329 (26.5)	297 (22.5)	192 (29.2)	<0.01
22G	2,182 (66.8)	797 (64.2)	951 (72.0)	434 (66.1)	<0.01
24G	59 (1.8)	19 (1.5)	24 (1.8)	16 (2.4)	0.44
Duration of catheter dwell^c^, median (IQR), hour	46.9 (22–83.6)	40 (20.6–74.4)	49.3 (23.4–89.2)	54.0 (24.1–89.5)	<0.01
Phlebitis (n,%)	302 (9.2)	117 (9.3)	130 (9.7)	55 (8.2)	0.56

APACHE, acute physiology and chronic health evaluation; ICU, intensive care unit; IQR, interquartile range; PIVC, peripheral intravascular catheter; SD, standard deviation.

Missing data: a, *n* = 674 (20.6%); b, *n* = 48 (1.5%); c = 9 (0.3%).

*PEU-Vialon^®^ is specified polyurethane.

**This value could not be calculated.

### Patient characteristics

The characteristics of the included patients are shown in Table 1. Overall, the mean age (SD) was 68.5 (15.2) years—819 patients (63.2%) were men, 449 patients (35.9%) were admitted to the ICU for cardiogenic disease, 633 patients (50.6%) needed mechanical ventilation within 24 h of admission to the ICU, 63 patients (5.0%) died in the ICU, and phlebitis occurred in 97 (7.8%) patients during the ICU stay. There were statistically significant differences for almost all variables in each group stratified using the APACHE II score. There was one (0.08%) missing data point for body height, two (0.2%) for body weight and BMI, and seven (0.6%) for ICU admission.

### Peripheral intravascular catheter characteristics

The characteristics of the included PIVCs are shown in Table 2. Overall, 3,219 catheters (98.5%) were inserted with the provision of standardized drug administration measures in the ICU; 2,313 catheters (70.8%) were inserted by the nurse; 1,752 catheters (53.6%) were inserted in the forearm; and 1,252 catheters (38.3%) were made of tetrafluoroethylene. The median duration of catheter dwell (IQR) was 46.9 h (22–83.6), and phlebitis occurred in 302 catheter cases (9.2%) during the ICU stay. There were statistically significant differences for almost all variables in each group stratified using the APACHE II score. There were 674 missing data points (20.6%) for the variable on medical staff inserting the catheter, 48 (1.5%) in the catheter gauge, and 9 (0.3%) in the duration of catheter dwell.

### Administered drug characteristics

The characteristics of the administered drugs are shown in Table 3. Amiodarone was administered in 41 catheters (1.3%), nitroglycerin in 57 catheters (1.7%), heparin in 309 catheters (9.5%), nicardipine in 290 catheters (8.9%), noradrenaline in 86 catheters (2.6%), and vancomycin in 117 catheters (3.6%). There was no missing data.

**TABLE 3:** Administered drug characteristics during insertion stratified using the APACHE II score*.

Variables (n,%)	Overall *n* = 3,267	Group 1 (APACHE II score ≤ 15) *n* = 1,260 (38.6%)	Group 2 (16–25 of APACHE II score) *n* = 1,339 (41.0%)	Group 3 (APACHE II score ≥ 26) *n* = 668 (20.4%)	*p* value
Albumin	169 (5.2)	46 (3.7)	69 (5.2)	54 (8.1)	<0.01
Amiodarone	41 (1.3)	7 (0.6)	22 (1.6)	12 (1.8)	0.02
Dobutamine	50 (1.5)	19 (1.5)	25 (1.9)	6 (0.9)	0.25
Fat	299 (9.2)	90 (7.1)	142 (10.6)	67 (10.0)	<0.01
Fentanyl	452 (13.8)	171 (13.6)	186 (13.9)	95 (14.2)	0.92
Nitroglycerin	57 (1.7)	17 (1.3)	31 (2.3)	9 (1.3)	0.12
Heparin	309 (9.5)	143 (11.3)	120 (9.0)	46 (6.9)	<0.01
Magnesium	105 (3.2)	44 (3.5)	39 (2.9)	22 (3.3)	0.70
Meropenem	132 (4.0)	24 (1.9)	69 (5.2)	39 (5.8)	<0.01
Midazolam	56 (1.7)	11 (0.9)	30 (2.2)	15 (2.2)	<0.01
Nicardipine	290 (8.9)	138 (11.0)	111 (8.3)	41 (6.1)	<0.01
Noradrenaline	86 (2.6)	20 (1.6)	43 (3.2)	23 (3.4)	0.01
Potassium	149 (4.6)	50 (4.0)	53 (4.0)	46 (6.9)	<0.01
Vancomycin	117 (3.6)	35 (2.8)	46 (3.4)	36 (5.4)	0.01

APACHE, acute physiology and chronic health evaluation.

*There was no missing data.

### Risk factors for phlebitis depending on the acute physiology and chronic health evaluation II score

Univariate and multivariable multilevel marginal Cox regression analyses were performed for each group stratified using the APACHE II score (Table 4 and Supplementary Table 4). After the multivariable multilevel marginal Cox regression analyses, there were significant differences in HR (95% CI) in several risk factors as follows: (i) in group 1, provision of standardized drug administration measures in the ICU (HR, 0.4 [0.17–0.9]; *p* = 0.03) and nicardipine administration (HR, 2.25 [1.35–3.75]; *p* < 0.01); (ii) in group 2, catheter insertion by the doctor using the nurse as a reference (HR, 0.48 [0.23–0.97]; *p* = 0.04), catheter insertion in the upper arm using the forearm as a reference (HR, 0.41 [0.2–0.83]; *p* = 0.01), use of PEU-Vialon^®^, a specified polyurethane catheter, using polyurethane as a reference (HR, 0.56 [0.34–0.92]; *p* = 0.02), amiodarone administration (HR, 6.33 [2.6–15.4]; *p* < 0.01), nicardipine administration (HR, 1.9 [1.16–3.12]; *p* = 0.01), and noradrenaline administration (HR, 3.0 [1.52–5.88]; *p* < 0.01); (iii) in group 3, noradrenaline administration (HR, 3.39 [1.14–10.1]; *p* = 0.03) (Table 4).

**TABLE 4:** Multivariable analysis for phlebitis using marginal Cox regression analysis stratified using the APACHE II score.

Variables	Group 1 (APACHE II score ≤ 15) *n* = 880 Phlebitis: *n* = 93 (10.6%)		Group 2 (16–25 of APACHE II score) *n* = 1,111 Phlebitis: *n* = 122 (11.0%)		Group 3 (APACHE II score ≥ 26) *n* = 588 Phlebitis: *n* = 44 (7.5%)	
			
	HR (95% CI)	*p* value	HR (95% CI)	*p* value	HR (95% CI)	*p* value
Age	1.01 (1.0–1.02)	0.18	1.01 (0.99–1.02)	0.39	1.0 (0.98–1.03)	0.8
Men	0.80 (0.5–1.26)	0.33	0.75 (0.52–1.1)	0.14	0.61 (0.32–1.17)	0.14
**BMI**						
18.5–25	ref	−	ref	−	ref	−
≤18.5	1.08 (0.53–2.18)	0.84	1.16 (0.74–1.82)	0.52	0.57 (0.13–2.47)	0.45
≥25	1.33 (0.83–2.13)	0.24	0.84 (0.53–1.34)	0.46	0.97 (0.49–1.92)	0.93
Provision of standardized drug administration measures in the ICU	0.4 (0.17–0.9)	0.03	0.66 (0.15–2.94)	0.59	–**	-**
**Medical staff inserting the catheter**						
Nurse	ref	−	ref	−	ref	−
Doctor	0.51 (0.22–1.19)	0.12	0.48 (0.23–0.97)	0.04	0.71 (0.27–1.9)	0.49
**Insertion site**						
Forearm	ref	−	ref	−	ref	−
Upper arm	0.57 (0.24–1.32)	0.19	0.41 (0.2–0.83)	0.01	1.07 (0.38–3.02)	0.9
Elbow	0.22 (0.03–1.58)	0.13	1.17 (0.52–2.62)	0.7	1.85 (0.52–6.6)	0.34
Wrist	0.2 (0.03–1.42)	0.11	0.58 (0.21–1.6)	0.29	3.94 (0.87–17.8)	0.08
Hand	0.46 (0.21–1.01)	0.05	0.62 (0.32–1.18)	0.29	2.0 (0.82–4.76)	0.13
Lower leg	0.9 (0.4–2.01)	0.79	0.73 (0.36–1.48)	0.39	1.92 (0.67–5.5)	0.23
Dorsal foot	1.32 (0.53–3.25)	0.55	0.55 (0.2–1.53)	0.25	2.24 (0.85–5.89)	0.1
**Catheter materials**						
Polyurethane	ref	−	ref	−	ref	−
PEU-Vialon^®^*	0.87 (0.5–1.52)	0.63	0.56 (0.34–0.92)	0.02	1.83 (0.67–5.01)	0.24
Tetrafluoroethylene	0.94 (0.53–1.68)	0.85	0.99 (0.64–1.52)	0.95	2.03 (0.78–5.26)	0.15
**Administered drug**						
Amiodarone	1.71 (0.23–12.9)	0.6	6.33 (2.6–15.4)	<0.01	0.98 (0.13–7.49)	0.99
Nitroglycerin	0.41 (0.06–2.97)	0.37	0.22 (0.03–1.63)	0.14	–***	0.99
Heparin	0.56 (0.27–1.16)	0.12	0.54 (0.25–1.2)	0.13	1.05 (0.34–3.22)	0.94
Nicardipine	2.25 (1.35–3.75)	<0.01	1.9 (1.16–3.12)	0.01	0.36 (0.05–2.67)	0.31
Noradrenaline	0.93 (0.21–4.04)	0.92	3.0 (1.52–5.88)	<0.01	3.39 (1.14–10.1)	0.03
Vancomycin	0.2 (0.03–1.46)	0.11	0.89 (0.28–2.85)	0.84	0.66 (0.15–2.84)	0.57

APACHE, acute physiology and chronic health evaluation; BMI, body mass index; CI, confidence interval; ER, emergency room; ICU, intensive care unit; IQR, interquartile range; HR, hazard ratio; PIVC, peripheral intravenous catheter.

*PEU-Vialon^®^ is specified polyurethane.

**This value could not be calculated since there was no catheter inserted without provision of standardized drug administration measures in the ICU.

***This value was almost zero and 95% CI was too wide to describe here.

## Discussion

In this study, patients were categorized into three groups based on a previous study using the APACHE II score as follows: ≤15 (group 1), 16–25 (group 2), and ≥26 (group 3) (6). There was phlebitis in 9.2% cases per catheter and no statistically significant difference between each group. After the multivariable, multilevel, marginal Cox regression analyses for each group, there were different risk factors for phlebitis in each group.

There are several possible reasons for the different risk factors for phlebitis found in each group. First, there might be different risk factors for phlebitis regarding administered drugs due to severity of the illness. Nicardipine administration was a statistically significant risk factor in group 1 and 2. In contrast, noradrenaline administration was a statistically significant risk factor in groups 2 and 3. Nicardipine was used more frequently in the following order: groups 1, 2, and 3. Meanwhile, noradrenaline was used more frequently in the following order: groups 3, 2, and 1. In addition, patients with septic shock were more frequently in the following order: groups 3, 2, and 1. Therefore, a more severe disease indicated lower blood pressure (i.e., nicardipine could not be administered; hence, noradrenaline was used); thus, there may be differences in the use of nicardipine and noradrenaline in each group. On the other hand, fat administration was used least frequently in group 1; however, it was not a statistically significant risk factor for phlebitis in a previous study (6). Therefore, since drugs administered at different frequencies in each group are not necessarily significant risk factors, there might be different risk factors for phlebitis regarding administered drugs due to severity of the illness. Second, the results of our study may have been influenced by bias in the distribution of the factors. Catheter insertion in the upper arm using the forearm as a reference was a significant risk factor in group 2. In this study, PIVCs were more frequently inserted in the forearm in groups 1 and 2. Meanwhile, PIVCs were less frequently inserted in the upper arm in groups 1 and 2. Therefore, catheter insertion in the upper arm using the forearm as a reference was not a statistically significant risk factor for phlebitis in groups 1 and 3. However, the sample size was not large enough to detect a significant difference for these variables, and there may be a significant difference in HR for these variables for all groups when more cases are accumulated.

We investigated the risk factors for peripheral phlebitis in patients stratified according to the severity of illness using the APACHE II score. Although some risk factors were common to all groups, those for phlebitis varied according to the severity of the illness. Therefore, clinicians should not focus on a single risk factor for phlebitis and should consider that various factors may become risk factors according to the severity of the illness during clinical management. By considering these different risk factors, different treatment options to avoid phlebitis may be provided according to the severity of the illness. For example, in critically ill patients with a high frequency of PIVC insertion, we might reduce the risk of phlebitis by increasing the frequency of drug administration via central venous catheters (CVCs). A previous study compared PIVC replacement every 72–96 h and as needed with the occurrence of phlebitis and reported no significant difference. However, this study did not describe the severity of the illness, and it is unclear whether the results can be indicated for critically ill patients (12). Therefore, critically ill patients may benefit from periodic PIVC replacement. However, for periodic PIVC replacement, an increase in the number of PIVC insertions may lead to an increased likelihood of phlebitis; hence, the use of CVCs may be considered.

Nevertheless, there are some limitations to this study. First, only the Japanese population was included in the study; since the physique of Japanese people is different from that of people of other nationalities, external validity may not be preserved. Overall, the mean BMI in this study was 22.9. Given that the average BMI in developed countries is approximately 25–30, the included patients would be considered thin compared with others (13). There is no previous study showing that BMI is a risk factor for phlebitis. However, there may be different results if different populations with larger BMIs are included. Second, the results of the analysis may be incorrect due to the insufficient presumed risk factors for phlebitis used in the multivariable, multilevel, marginal Cox regression analyses. As described in the section on statistical analysis, the presumed risk factors for phlebitis used in this analysis were extracted using multiple criteria and considering previous studies and clinical importance. In particular, the third criteria (p values calculated in a previous study for phlebitis were <0.1) may possibly be arbitrary. Therefore, if statistical analysis was performed using presumed risk factors for phlebitis other than those used in this study, the results may be different. Third, the results may have been unstable because of the large number of variables used to adjust for confounding factors in the multivariable, multilevel, marginal Cox regression analyses compared to the number of phlebitis occurrences. However, since the objective of this study was not to investigate a causal relationship between each factor and phlebitis but to show that the risk factors differed with the severity of the disease, we believe that the number of variables did not have a significant effect on the results. Finally, in treating the administered drug as a binary variable for the multivariable, multilevel, marginal Cox regression analyses, the risk of the drugs may have been underestimated and a statistically significant difference could not be detected. The effect of drugs depends not only on their administration but also on their dose. In the analysis, since both high- and low-dose drugs were treated as binary variables in the same category, the effect of the drugs may have been underestimated. Therefore, the results may differ if the drugs were treated as a continuous variable.

## Conclusion

We found that the risk factors for phlebitis varied according to the severity of illness. Taking into consideration these risk factors, different treatments may be provided to avoid phlebitis based on the patient’s severity of illness.

## Data availability statement

The raw data supporting the conclusions of this article will be made available by the authors, without undue reservation.

## Ethics statement

The studies involving human participants were reviewed and approved by Jichi Medical University Bioethics Committee for Clinical Research, Saitama Medical Center. Written informed consent for participation was not required for this study in accordance with the national legislation and the institutional requirements.

## Author contributions

YK and HY conceived, designed, and performed the analysis. YK interpreted the results and drafted the manuscript. HY, TM, MKa, MKo, YK, NK, KS, NS, KM, and TA provided revisions to the manuscript. All authors read and approved the final manuscript.

## Abbreviations

APACHE: acute physiology and chronic health evaluation
BMI: body mass index
CI: confidence interval
HR: hazard ratio
ICU: intensive care unit
IQR: interquartile range
PIVC: peripheral intravascular catheter
SD: standard deviations
STROBE: strengthening the reporting of observational studies in epidemiology.
