# Supplementary material for: A study of the risk factors for phlebitis in patients stratified using the acute physiology and chronic health evaluation II score and admitted to the intensive care unit: A post hoc analysis of the AMOR-VENUS study
Source: Front Med (Lausanne). 2022 Dec 5;9:965706. doi: 10.3389/fmed.2022.965706 (PMC9760669; doi:10.3389/fmed.2022.965706)
Supplement: Supplementary file 1 [file Data_Sheet_1.docx]

**Definition and measurement of phlebitis**

A blinded assessor in the main institution diagnosed phlebitis and classified it into four grades based on the criteria comprising six clinical signs (Supplementary Tables 2 and 3). In the case of patients with disturbed consciousness, well-trained nurses assessed the grade of symptoms, including pain, using face and behavioral pain scales^1^. Pilot training was implemented to gain an accurate phlebitis diagnosis and reduce information bias. Furthermore, well-trained expert clinician researchers at the central institution monitored the accuracy of phlebitis diagnosis during the study period. During the first month after beginning data collection, the data management center confirmed the accuracy of the information on the catheter insertion sites with phlebitis images sent from each institution.

**References**

1. Infusion Nurses Society. Infusion nursing standards of practice. J Infus Nurs (2006) 29:S1-92. doi: 10.1097/00129804-200601001-00001.

**Supplementary Table 2. The definition of phlebitis according to the Infusion Nurses Society (INS)**

| Grade | Definition |
| --- | --- |
| 0 | No clinical signs. |
| 1 | Erythema at the puncture site regardless of the presence or absence of pain. |
| 2 | Pain at the puncture site with erythema and/or edema. |
| 3 | Pain at the puncture site with erythema and/or edema and streak formation or a palpable venous cord. |
| 4 | Pain at the puncture site with erythema and/or edema and streak formation or a palpable venous cord > 1 inch. Presence of purulent drainage. |

**Supplementary Table 3. The definition of each variable for phlebitis according to the Infusion Nurses Society (INS)**

| Variable | Definition |
| --- | --- |
| Pain | Pain around the PIVC insertion site. |
| Erythema | Erythema around the PIVC insertion site. |
| Edema | Swelling around the PIVC insertion site. |
| Streak formation | Erythema along the blood vessels from the PIVC insertion site. |
| Palpable venous cord | Induration along the blood vessels from the PIVC insertion site. |

Abbreviation: PIVC, peripheral intravenous catheter.

**Supplementary Table 4. Univariate analysis for phlebitis using marginal Cox regression analysis stratified by APACHE Ⅱ score**

| **Variables** | **Group 1 (APACHE Ⅱ score: ≤ 15)**  **n = 1,260**  **Phlebitis: n = 117 (9.3%)** | | **Group 2 (16–25 of APACHE Ⅱ score)**  **n = 1,339**  **Phlebitis: n = 130 (9.7%)** | | **Group 3 (APACHE Ⅱ score ≥ 26)**  **n = 668**  **Phlebitis: n = 55 (8.2%)** | |
| --- | --- | --- | --- | --- | --- | --- |
|  | **HR (95% CI)** | **p value** | **HR (95% CI)** | **p value** | **HR (95% CI)** | **p value** |
| Age | 1.01 (1.0–1.03) | 0.02 | 1.0 (0.99–1.02) | 0.71 | 1.01 (0.98–1.03) | 0.61 |
| Male sex | 0.77 (0.54–1.11) | 0.16 | 0.76 (0.54–1.08) | 0.12 | 0.49 (0.29–0.83) | < 0.01 |
| BMI  18.5–25  ≤ 18.5  ≥ 25 | Ref  1.02 (0.56–1.85)  1.34 (0.9–2.0) | -  0.95  0.14 | Ref  1.15 (0.74–1.78)  0.88 (0.57–1.36) | -  0.55  0.56 | Ref  0.67 (0.2–2.16)  0.86 (0.48–1.53) | -  0.5  0.6 |
| Provision of standardized drug administration measures in the ICU | 0.31 (0.15–0.64) | < 0.01 | 0.52 (0.17–1.65) | 0.27 | -^**^ | -^**^ |
| Medical staff inserting the catheter  Nurse  Doctor | Ref  0.42 (0.18–0.96) | -  0.04 | Ref  0.59 (0.31–1.13) | -  0.11 | Ref  0.83 (0.33–2.12) | -  0.7 |
| Insertion site  Forearm  Upper arm  Elbow  Wrist  Hand  Lower leg  Dorsal foot | Ref  0.77 (0.35–1.67)  0.15 (0.02–1.08)  0.51 (0.19–1.4)  0.38 (0.2–0.72)  0.78 (0.36–1.68)  1.3 (0.6–2.82) | -  0.5  0.06  0.19  < .01  0.52  0.51 | Ref  0.58 (0.3–1.12)  0.87 (0.4–1.89)  0.52 (0.19–1.41)  0.51 (0.27–0.95)  0.69 (0.35–1.38)  0.56 (0.2–1.52) | -  0.11  0.73  0.2  0.03  0.3  0.25 | Ref  0.78 (0.29–2.07)  2.32 (0.87–6.19)  2.55 (0.76–8.59)  1.89 (0.9–3.96)  1.46 (0.55–3.88)  1.8 (0.72–4.49) | -  0.62  0.09  0.13  0.09  0.45  0.21 |
| Catheter materials  Polyurethane  PEU-Vialon^®*^  Tetrafluoroethylene | Ref  0.65 (0.42–1.0)  0.71 (0.45–1.11) | -  0.05  0.13 | Ref  0.46 (0.29–0.73)  0.9 (0.61–1.32) | -  < 0.01  0.58 | Ref  1.27 (0.56–2.88)  1.44 (0.68–3.04) | -  0.57  0.34 |
| Administered drug  Amiodarone  Glycerin  Heparin  Nicardipine  Noradrenaline  Vancomycin | 1.31 (0.18–9.39)  0.75 (0.19–3.05)  0.57 (0.31–1.03)  2.46 (1.6–3.79)  1.47 (0.47–4.63)  0.15 (0.02–1.07) | 0.79  0.69  0.06  < 0.01  0.51  0.06 | 5.01 (2.34–10.8)  0.47 (0.12–1.89)  0.54 (0.27–1.06)  2.04 (1.29–3.22)  2.66 (1.39–5.07)  0.58 (0.19–1.84) | < 0.01  0.29  0.07  < 0.01  < 0.01  0.36 | 0.74 (0.1–5.4)  0.84 (0.12–6.1)  1.11 (0.47–2.62)  0.29 (0.04–2.11)  3.6 (1.43–9.0)  0.47 (0.12–1.94) | 0.79  0.86  0.8  0.22  < 0.01  0.3 |

Abbreviations: APACHE, Acute Physiology and Chronic Health Evaluation; BMI, body mass index; CI, confidence interval; ER, emergency room; ICU, intensive care unit; IQR, interquartile range; HR, hazard ratio; PIVC, peripheral intravenous catheter.

^*^PEU-Vialon^®^ is specified polyurethane.

^**^This value could not be calculated since there was no catheter inserted without provision of standardized drug administration measures in the ICU.
